# New Semisynthetic Penicillins Obtained by Coupling of the 6-Aminopenicillanic Acid with 5-Mercapto-1,2,4-triazoles-3,4-disubstituted

**DOI:** 10.3390/ijms24021497

**Published:** 2023-01-12

**Authors:** Corina Cheptea, Alexandru Zara, Dan Gheorghe Dimitriu, Valeriu Sunel, Dana Ortansa Dorohoi, Toni Andor Cigu

**Affiliations:** 1 Department of Biomedical Sciences, Faculty of Medical Bioengineering, “Grigore T. Popa” University of Medicine and Pharmacy, 9-13 M. Kogalniceanu Str., RO-700454 Iasi, Romania; 2 Faculty of Physics, Alexandru Ioan Cuza University, 11 Carol I Blvd., RO-700506 Iasi, Romania; 3 Faculty of Chemistry, Alexandru Ioan Cuza University, 11 Carol I Blvd., RO-700506 Iasi, Romania

**Keywords:** semisynthetic penicillins, triazole derivatives, indazole, antimicrobial activity

## Abstract

In a basic medium, 5-Mercapto-1,2,4-triazoles pass into the thiol form, allowing their transformation into sodium salts, which, in reaction with sodium monochloroacetate, lead to sodium 5-thioacetates of 1,2,4-triazoles-3,4-disubstituted. Sulfur derivatives converted to pivalic mixed anhydrides were used as active forms in the acylation of 6-amino penicillanic acid (6-AP) to obtain new semisynthetic penicillins. They contain in the molecule, together with the β-lactam ring, the nucleus 3-[(5-nitroindazol-1′-yl-methyl)]-4-aryl-5-mercapto-1,2,4-triazole, both contributing to an important antibacterial effect. The structure of the new antibiotics was confirmed by the results of elemental and spectral analysis (FT-IR, ^1^H- and ^13^C-NMR). The synthetic penicillins were tested for toxicological action and antibacterial activity and the obtained results were close to those for amoxicillin, the reference drug.

## 1. Introduction

The problem of obtaining new semisynthetic penicillins is today the concern of many research teams. This concern aims to achieve the desideratum that emerged after so many years of penicillin therapy, regarding both antimicrobial and pharmacological properties: stability to penicillinase, broader antibacterial spectrum, stability to acids, and absence of side effects [1,2,3,4].

**Scheme 1:**
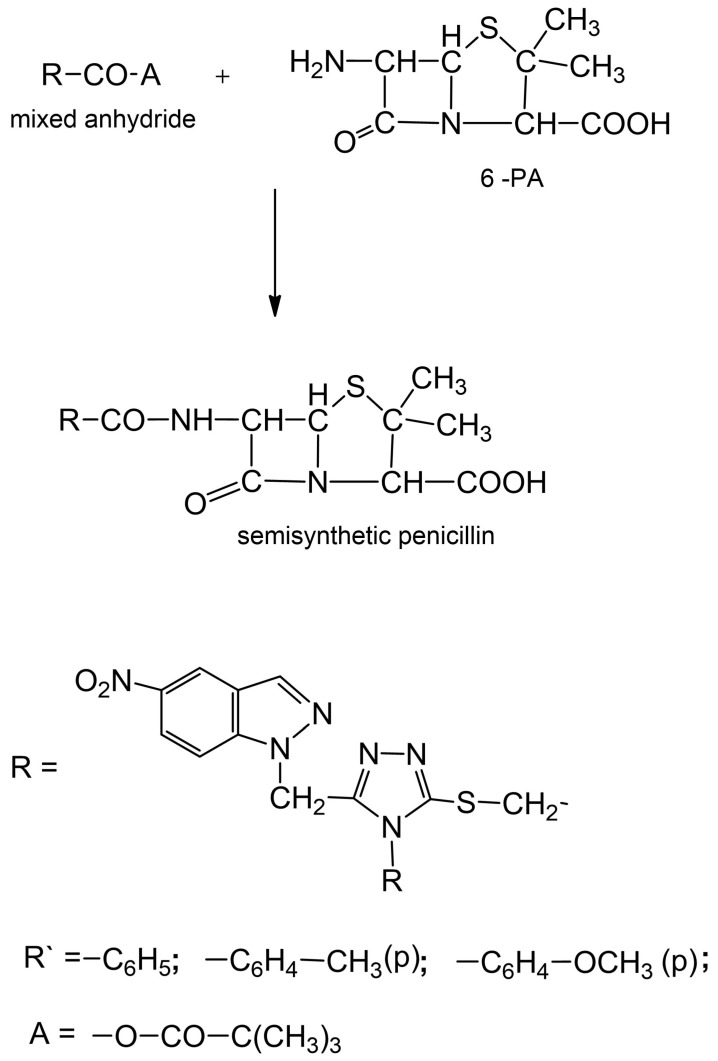
Acylation of 6-aminopenicillanic acid (6-PA) with 5-mercapto-1,2,4-triazole-disubstituted derivatives.

In this sense, the semisynthesis offers much wider possibilities than biosynthesis, even if this is directed. The basic product in semisynthesis is 6-aminopenicillanic acid. When this is treated with acid chloride [5,6,7] or mixed anhydride [2,3,8] under certain conditions, the 6-acyl-aminopenicilanic acid is obtained, a semisynthetic penicillin. The acylation of 6-aminopenicillanic acid with 5-mercapto-1,2,4-triazole-disubstituted derivatives in active forms follows the general scheme shown in Scheme 1.

This research focuses on the semisynthesis of penicillins, starting from 5-mercapto-1,2,4-triazoles-3,4-disubstituted.

The synthesis of this type of penicillin is interesting because, on the one hand, the presence of the triazole nucleus in the molecule of some substances impresses new qualities, which make them applicable in various domains of medical practice, and on the other hand, the nature of the substituent or its position in the molecule determines new pharmacological properties: antibacterial [9,10,11,12,13], antifungal [12,14,15], anti-inflammatory [10,16,17,18], analgesic-antipyretic [10,19,20,21], cardioprotective [22], or antitumor [11,23].

The 1,2,4-triazole derivatives used by us have a special feature, namely, they are substituted in four positions with a free phenyl residue, containing in turn various substituents in the para-position (see Scheme 1). By obtaining and characterizing new penicillins as having a “side chain” of 5-mercapto-1,2,4-triazole-3,4 disubstituted derivatives, both antibacterial and pharmacological properties were pursued, such as the stability to penicillinase (and consequently the activity against germs resistant to natural penicillins), a wider antibacterial spectrum (gram-negative germs), stability to acids (hence the possibility of oral administration), and the absence of allergic effects.

## 2. Results and Discussion

### 2.1. Chemical Synthesis

The synthesis of the new penicillins was performed by the reaction between sodium 1,2,4-triazole-5-α-thioacetate derivatives in activated form, with 6-aminopenicillanic acid.

The triazole derivatives (Ia, Ib, and Ic) were obtained by basic cyclization of 1-(5′-nitroindazol-1′-yl-acetyl)-4-aryl-thiosemicarbazides prepared in turn by condensation of aromatic isothiocyanates with 5-nitroindazol-1-yl-acetic acid hydrazide [24].

**Scheme 2:**
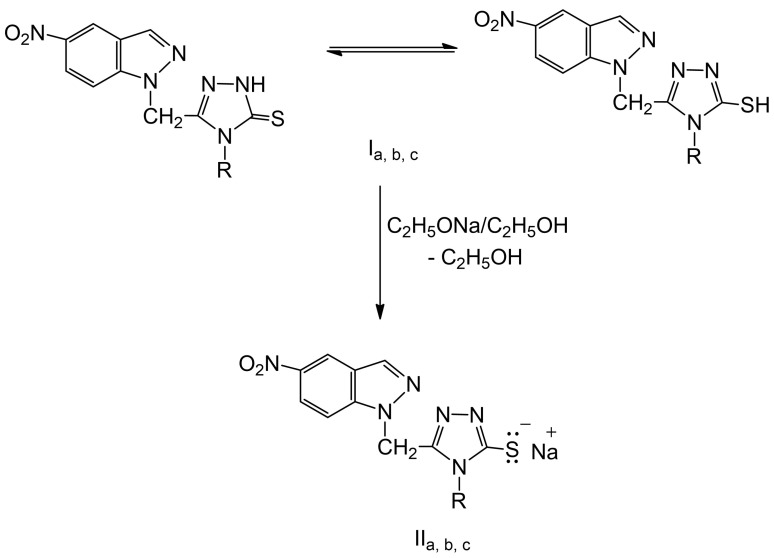
Preparation of 3-[(5′-nitro-1H-indazol-1′-yl) methyl]-N-aryl-1,2,4-triazole-5-thione sodium salts (IIa–IIc). The substituents are: R = –C_6_H_5_ for Ia, IIa; R = –C_6_H_4_–CH_3_ (p) for Ib, IIb; and R = –C_6_H_4_-OCH_3_ (p) for Ic, IIc.

The 5-mercaptotriazoles (Ia–Ic) present in the basic environment the phenomenon of double reactivity, a property that explains the existence of the reaction center at the sulphur atom in position five of the triazole heterocycle. This property made it possible for the conversion of the compounds Ia–Ic to sodium salts IIa–IIc by refluxing in anhydrous ethanol containing sodium ethoxide (Scheme 2).

After washing with anhydrous acetone and drying, the sodium mercaptides (IIa–IIc) are obtained in microcrystalline, in brown or reddish-brown form, having high melting points and yield in the finished product between 84 and 90%.

The structure of the derivatives (IIa–IIc) was established by chemical elemental analysis and spectral analysis (FT-IR, ^1^H- and ^13^C-NMR).

Thus, in the FT-IR spectra, the band characteristic of the C–S–Na group is present at 628–651 cm^−^^1^ and the absorption of the C = N group determines the appearance of some bands at 1613–1689 cm^−^^1^. The NO_2_ group corresponds to two absorption bands, symmetrical at 1335–1342 cm^−^^1^ and asymmetrical at 1516–1518 cm^−^^1^. The bands corresponding to the substituted benzene nucleus have been also identified in the range 793–897 cm^−^^1^.

The ^1^H-NMR spectra of compounds (IIa–IIc) show the protons of the CH_2_ group as a singlet in the region 5.72–5.74 ppm, while the aromatic protons appear as a doublet at 6.73–7.51 ppm and as a singlet at 8.32–8.36 ppm. The protons of the methyl group in the mercaptides IIb and IIc are found as a singlet at 2.15 ppm and 3.62 ppm, respectively.

**Scheme 3:**
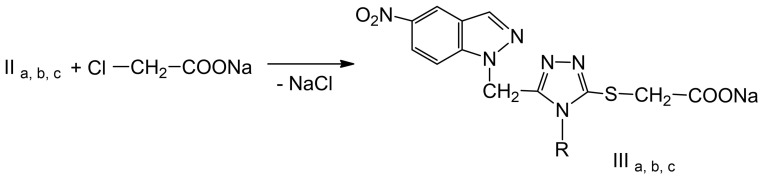
The synthesis of 3-[(5′-nitro-1H-indazol-1′-yl)-methyl]-N-aryl-1,2,4-triazol-5-sodium thioacetates (IIIa–IIIc). The substituents are: R = –C_6_H_5_ for IIa, IIIa; R = –C_6_H_4_–CH_3_ (p) for IIb, IIIb; and R = –C_6_H_4_-OCH_3_ (p) for IIc, IIIc.

The mercapto-triazoles in the –SNa form (IIa–IIc) react with sodium monochloroacetate in an anhydrous ethyl alcohol medium, forming five derivatives (IIIa–IIIc) (Scheme 3).

The structure of sodium thioacetates (IIIa–IIIc) was determined by chemical and spectral analysis (FT-IR, ^1^H- and ^13^C-NMR).

In the FT-IR spectra, the frequencies of the symmetric and asymmetric vibrations of the NO_2_ group are in the ranges 1338–1346 cm^−^^1^ and 1514–1519 cm^−^^1^, respectively, while the carboxylated ion is identified at 1616 cm^−^^1^, 1617 cm^−^^1^. The frequency of the S–CH_2_ bond is between 1420 cm^−^^1^ and 1496 cm^−^^1^, while distinct absorptions occur at 1673–1678 cm^−^^1^, corresponding to the C = N bond.

In the ^1^H-NMR spectra, the protons of the CH_2_ group from the 1′ position of the indazole nucleus appear as a singlet in the region 5.61–5.67 ppm, while the protons of the CH_2_ group bound to the sulphur atom could be detected, also as a singlet, in the region 4.38–4.49 ppm. The protons of the methyl group in compounds IIIb and IIIc are identified as singlets at 2.19 ppm and 3.39 ppm, respectively, while the aromatic protons appear for the compounds IIIa–IIIc as a doublet at 6.73–7.51 ppm and as a singlet at 8.32–8.36 ppm.

For the grafting of the triazole ring on the nucleus of 6-aminopenicillanic acid, it was necessary to activate the carboxylic function of sodium thioacetates (IIIa–IIIc), transforming them into mixed type A anhydrides, able to rapidly and mildly react with the amine group of β-lactamic acid. The pivaloyl chloride is an ideal partner of this transformation, which, for electronic and steric reasons of the three methyl groups at the same carbon atom, facilitates the cleavage of the mixed anhydride at one of the two carbonyls in the desired direction [25,26].

**Scheme 4:**
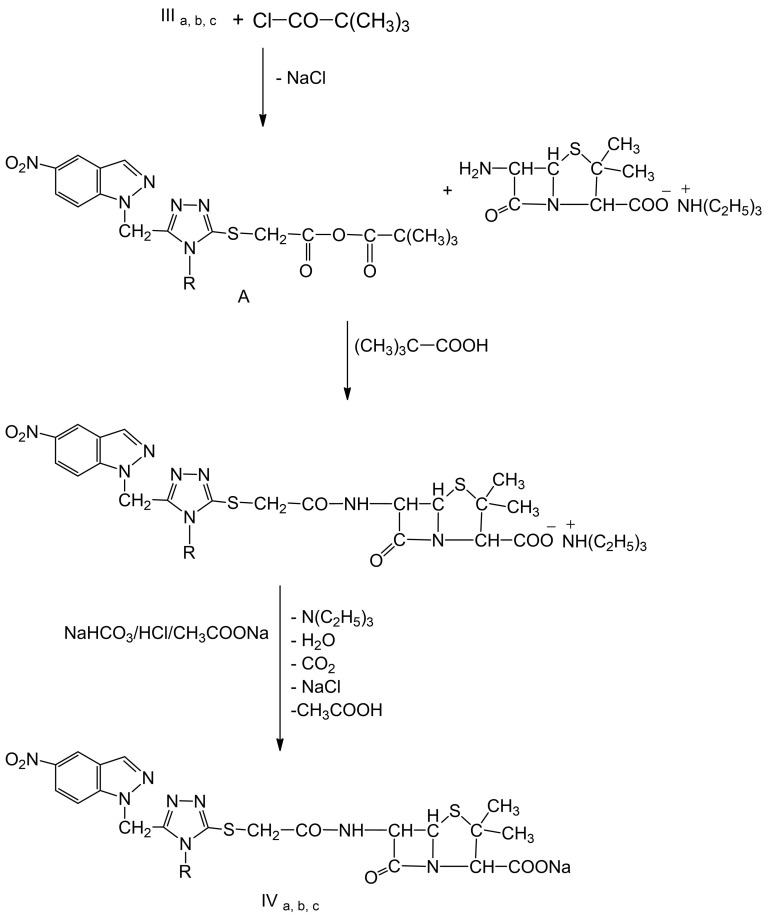
Obtaining and isolating the 3-[(5′-nitro-1H-indazol-1′-yl)-methyl] -N-aryl-1,2,4-triazole-5-thioacetamido-sodium penicillinate (IVa–IVc). The substituents are: R = –C_6_H_5_ for IIIa; IVa, R = –C_6_H_4_–CH_3_ (p) for IIIb, IVb; and R = –C_6_H_4_-OCH_3_ (p) for IIIc, IVc.

The mixed anhydrides (type A) are unstable and difficult to isolate, so the option was for their use “in situ” in dichloromethane solution, in which the 6-aminopenicillanic acid is dissolved in the form of triethylammonium salt. After completion of the coupling reaction, the dichloromethane is removed in vacuo and the distillation residue is dissolved in saturated sodium bicarbonate solution, which is when penicillin passes into an alkaline solution as a sodium salt. By acidifying the bicarbonate solution to pH = 1.5–2 in the presence of butyl acetate, the acid penicillin passes into the organic layer, from where it is isolated as a sodium salt (IVa–IVc) by precipitation with sodium acetate or sodium ethyl hexanoate (Scheme 4).

The penicillins were purified by dissolving them in distilled water and extracting butyl acetate from acidic penicillin after acidification, followed by the re-precipitation of sodium salts. Their purification was applied until a constant value of rotation [α] D was reached. The sodium salts of these penicillins are in the form of white microcrystals, stable at room temperature. All of them have a characteristic odor of penicillin and are easily soluble in water.

The chemical structure of β-lactam antibiotics (IVa–IVc) was confirmed by elemental and spectral analysis (FT-IR, ^1^H-, and ^13^C-NMR).

The FT-IR spectra indicate the presence of the lactam group C=O at 1702–1790 cm^−^^1^, while the bands corresponding to the valence vibration of the amide bond C=O appear at 1616–1677 cm^−^^1^. The NH amide groups are indicated by the presence of bands at 3120–3312 cm^−^^1^, while the bands attributed to the symmetric and asymmetric vibration of the NO_2_ group appear at 1337–1372 cm^−^^1^ and 1515–1534 cm^−^^1^, respectively. The para-disubstituted aromatic nucleus determines the appearance of some absorptions at 798 cm^−^^1^, 816 cm^−^^1^, and 1067 cm^−^^1^, while the COO^–^ group is assigned to bands from 1596 to 1618 cm^−^^1^.

In the ^1^H-NMR spectra, the characteristic signals of protons from the penicillin cycle appear at values of δ = 4.15–4.59 ppm, while the presence of the CH_3_ groups related to the penicillin cycle gives signals in the form of a singlet, at δ = 1.44–1.51 ppm. The protons in the NH amide groups occur at values of δ = 9.01 ppm, δ = 9.13 ppm, and δ = 9.17 ppm. Between 7.13–7.81 ppm and 8.19–8.36 ppm, there are signals attributed to the aromatic protons, while at 5.16–5.41 ppm and 3.71–3.93 ppm there is the singlet characteristic of the CH_2_ group from 1′ position of the indazole heterocycle, the singlet of the CH_2_ group related to the atom of sulphur.

In the ^13^C-NMR spectra, signals for the C=O appear at 171.33–177.87 ppm, for C-S at 65.35–145.80 ppm, and for the C-N group at 91.56–148.79 ppm. Signals for aromatic carbon at 111.51–148.13 ppm, for CH_3_ groups at 25.62–56.79 ppm, and for CH_2_ groups at 41.32–54.83 ppm also appear in the spectra.

### 2.2. Biological Activity Testing

#### 2.2.1. Evaluation of Penicillins IVa–IVc Toxicity

The acute toxicity of a substance consists in the evaluation of the mortality produced by its administration in a certain way, usually intraperitoneal or oral. Quantitatively, this is expressed by the lethal dose *LD*_50_, which causes death in 50% of the animals on which the experiment was performed.

The degree of toxicity was determined using white mice weighing 20 ± 2 g.

The animals were kept under observation for 7 days at room temperature (22 °C ± 1 °C), receiving standard food and water. Their weight was determined by periodic weighing every two days and at the same time. The animals that lost weight were removed.

Semisynthetic penicillins IVa–IVc and amoxicillin (reference drug) were dissolved/suspended in Tween 80 and administered intraperitoneally, lethality being recorded at 14 days.

The *LD*_50_ was thus determined using the Spearman–Kärber method [27]. The obtained results are listed in Table 1, Table 2, Table 3, Table 4 and Table 5.

By using Equation (1), the values of *LD*_50_ were calculated, the results being shown in Table 5.

The standard deviation was estimated from Equation (1) by the logarithmic method as being 1200 mg/kg body weight.

The obtained results, comparable to the *LD*_50_ value for amoxicillin (reference drug), show that penicillins IVa–IVc have a toxicity that falls within acceptable limits, recommending them for laboratory screening.

#### 2.2.2. Testing of the Antimicrobial Activity of Penicillins IVa–IVc

The evaluation of the antimicrobial capacity of the compounds IVa–IVc was performed in order to establish a correlation between their biological activity and their chemical structure.

The antimicrobial tests were performed in the Microbiology Laboratory, Epidemiology Section of the Institute of Public Health (IPH) from Iasi.

The gram-positive and gram-negative germs from the collection of the Microbiology Laboratory of IPH were prepared in advance, regenerating from the storage medium, after which their morphological, tincture, and culture characteristics were verified: solids Mueller–Hinton, blue agar-bromothymol-lactose, agar-blood, as well as on the liquid medium nutritious simple meat broth. Specifically, the gram-positive *Streptococcus pneumoniae* (ATCC-46619) was grown on Mueller–Hinton agar with 5% sheep blood. The incubation time was 20 h at 35 °C, in the presence of 5% CO_2_.

The metabolic characteristics were monitored for the mediums Triple-Sugar-Iron agar, Mobility-Indole-Urea, Motility-Indole-Lysine Decarboxylase, and Phenylalanine deaminase and confirmed by APJ-20-Easystem kits.

All tested strains showed fully expressed phenotypic characteristics, being pure and qualitatively suitable for use in the experiments.

Bacterial cultures were prepared for testing using suspensions with a concentration of 1.5 × 10^8^ colony forming units/mL, the density of 0.5 McFarland being determined with a densitometer (Biosan DEN-1B).

Both gram-positive germs *Streptococcus pneumoniae* (ATCC-46619), *Staphylococcus aureus* (ATCC-25923) and gram-negative germs *Klebsiella pneumoniae* (C-82), *Pseudomonas aeruginosa* (ATCC-27853), *Escherichia coli* (ATCC-25922) were used to test the antimicrobial activity of β-lactam antibiotics IVa–IVc.

Amoxicillin, known for its antimicrobial activity against certain gram-positive and gram-negative germs [28,29,30], was used as a reference substance.

The microbial strains, as a suspension in saline, were cultured on Mueller–Hinton agar and incubated at 37 °C for 18 h.

The IVa–IVc semisynthetic penicillins were dissolved in Tween 80 (100 μg/mL Tween 80), after which the resulting solutions were introduced into the culture medium (100 mL/sample).

The same was performed with the reference antibiotic (amoxicillin). Sterilization was performed at 120 °C for 20 min. In parallel, a control test was performed, only with Tween 80, to highlight the possible activity of the solvent on the test germs.

All operations were performed in the sterile box. The assessment of antimicrobial activity was performed by the Kirby–Bauer method [31], the readings being made at 24 h. The results of the antimicrobial tests were expressed, for compounds IVa–IVc, by the diameter of the inhibition zone (see Table 6).

The preliminary test by the diffusimetric method indicates that for the compound IVa, which contains in the molecule an unsubstituted phenyl nucleus, there is a very small spectrum of antimicrobial action. In the case of penicillins IVb and IVc, the antimicrobial action of the product containing the p-methoxyphenyl group in the molecule IVc is higher than that of penicillin IVb, which contains the p-tolyl group.

It follows that the antibacterial spectrum of the new penicillins is influenced by the structure of the existing substituent on the nitrogen atom at position four of the triazole nucleus in the side chain of penicillins IVa–IVc.

At the same time, it is found that the penicillins IVb and IVc are sensitive to the test germs used, the closer one to the biological activity of amoxicillin being penicillin IVc.

For penicillins IVb and IVc, the minimum inhibitory concentration (MIC) was also determined by the microdilution method [32], diluting the stock solution (100 μg antibiotic/mL Tween 80) so as to obtain solutions with different concentrations, which were subsequently introduced in the culture medium. The obtained values of the minimum inhibitory concentration are presented in Table 7.

The determination of the minimum inhibitory concentration shows that the values recorded in the case of penicillin IVc are close to those recorded for the reference compound (amoxicillin) [33]. Moreover, both penicillins IVb and IVc inhibit the gram-negative germ ***Pseudomonas aeruginosa***, while the reference compound is inactive.

## 3. Materials and Methods

### 3.1. General

The chemical reactants were purchased from both Merck Company and Fluka (Sigma-Aldrich, St. Louis, MO, USA) and used without any purification.

The purity of the obtained substances was checked by quantitative elemental analysis, as well as Fourier-transform infrared (FT-IR) and nuclear magnetic resonance (NMR) spectroscopy. Quantitative elemental analysis was performed by using the Exeter Analytical CE 440. BRUKER Tensor-27 FT-IR (ATR) spectrophotometer was used to record the FT-IR spectra. ^1^H- and ^13^C-NMR spectra (DMSO-d_6_, 400 MHz and 100 MHZ, respectively) were recorded using a BRUKER ARX 400 spectrometer equipped with 5 mm QNP ^1^H/^13^C/^31^P/^19^F samples and Silicon Graphics INDIGO^2^ workstation.

### 3.2. Chemical Compounds

**3-[(5′-nitroindazol-1′-yl)-methyl)]-4-aryl-5-mercapto-1,2,4-triazoles (Ia–Ic)**

These were synthesized by cyclization of the corresponding 1-(5’-nitroindazol-1′-yl-acetyl)-4-aryl-thiosemicarbazides in alkaline solution [24].

**3-[(5′-nitro-1H-indazol-1′-yl)-methyl]-N-aryl-1,2,4-triazole-5-sodium mercaptides (IIa–IIc)**

In a reaction flask provided with reflux cooler, 50 mL of anhydrous ethyl alcohol and 0.025 mol metallic sodium were added in several portions, under continuous stirring. Over the hot sodium ethoxide solution, 0.025 mol of 5-mercapto-1,2,4-triazole-3,4-disubstituted derivative (Ia–Ic) was added, after which the reaction mixture was refluxed for 90 min. The excess alcohol was removed by distillation under reduced pressure, and the sodium salts were filtered in vacuo and dried.

For purification, each of the sodium mercaptides (IIa–IIc) was dissolved in 50 mL of anhydrous ethyl alcohol, filtered, and then each sodium salt was reprecipitated from the filtrate by adding 70 mL of dioxane, previously cooled. After filtering, the products were dried in a vacuum oven at 40–50 °C and conditioned into a well-closed glass bottle.

**3-[(5′-nitro-1H-indazol-1′-yl)-methyl]-N-phenyl-1,2,4-triazole-5-sodium mercaptide (IIa)**

Orange-brown, crystalline solid (7.85 g; yield 84%); m. p. = 251–253 °C.

Anal. calc. for C_16_H_11_N_6_O_2_SNa (%): C, 51.33; H, 2.94; N, 22.45; S, 8.55. Found (%): C, 51.60; H, 3.13; N, 22.81; S, 8.95.

FT-IR; γ_max_ (cm^−^^1^): 1342 (NO_2_ symm); 1576 (NO_2_ assym); 1681 (C=N); 630 (C–SNa); 821 (monosubstituted benzene nucleus).

^1^H-NMR (DMSO-d_6_, 400 MHz), δ (ppm): 5.72 (s, 2H, CH_2_); 7.10–7.12 (d, *J* = 9.2 Hz, 2H, CHAr); 7.21–7.22 (d, *J* = 6.8 Hz, 2H, CHAr); 7.48–7.50 (t, 1H, CHAr); 7.71–7.73 (d, *J* = 8.4 Hz, 1H, CHAr); 8.09 (s, 1H, CHAr); 8.16 (s, 1H, CHAr); 8.20–8.22 (d, *J* = 7.2 Hz, 1H, CHAr).

**3-[(5′-nitro-1H-indazol-1′-il)-methyl]-N-(p-tolyl)-1,2,4-triazole-5-sodium mercaptide (IIb)**

Red-brown, crystalline solid (8.53 g; yield 88%); m.p. = 252–254 °C.

Anal. calc. for C_17_H_13_N_6_O_2_SNa (%): C, 52.57; H, 3.35; N, 21.64; S, 8.24. Found (%): C, 52.79; H, 3.51; N, 21.95; S, 8.59.

FT-IR; γ_max_ (cm^−^^1^): 1335 (NO_2_ symm); 1517 (NO_2_ assym); 1613 (C=N); 628 (C–SNa); 897 (para-disubstituted benzene nucleus).

^1^H-NMR (DMSO-d_6_, 400 MHz), δ (ppm): 2.15 (s, 3H, CH_3_); 5.74 (s, 2H, CH_2_); 6.95–6.97 (d, *J* = 9.6 Hz, 2H, CHAr); 7.02–7.04 (d, *J* = 9.6 Hz, 2H, CHAr); 7.49–7.53 (d, *J* = 14.8 Hz, 1H, CHAr); 7.73–7.75 (d, *J* = 10.4 Hz, 1H, CHAr); 8.29 (s, 1H, CHAr); 8.84 (s, 1H, CHAr).

**3-[(5′-nitro-1H-indazol-1′-il)-methyl]-N-(p-methoxyphenyl)-1,2,4-triazole-5-sodium mercaptide (IIc)**

Dark-brown, crystalline solid (9.09 g; yield 90%); m.p. = 233–235 °C.

Anal. calc. for C_17_H_13_N_6_O_3_SNa (%): C, 50.49; H, 3.21; N, 20.79; S, 7.92. Found (%): C, 50.68; H, 3.43; N, 21.11; S, 8.20.

FT-IR; γ_max_ (cm^−^^1^): 1340 (NO_2_ symm); 1518 (NO_2_ assym); 1615 (C=N); 651 (C–SNa); 793 (para-disubstituted benzene nucleus).

^1^H-NMR (DMSO-d_6_, 400 MHz), δ (ppm): 3.62 (s, 3H, OCH_3_); 5.73 (s, 2H, CH_2_); 6.73–6.75 (d, *J* = 9.2 Hz, 2H, CHAr); 6.98–6.99 (d, *J* = 5.6 Hz, 2H, CHAr); 7.49–7.51 (d, *J* = 9.6 Hz, 1H, CHAr); 8.06–8.08 (d, *J* = 9.2 Hz, 1H, CHAr); 8.32 (s, 1H, CHAr); 8.36 (s, 1H, CHAr).

**3-[(5′-Nitro-1H-indazol-1′-yl)-methyl]-N-aryl-1,2,4-triazol-5-α- sodium thioacetates (IIIa–IIIc)**

The 0.02 mol compound (IIa–Iic) was dissolved in 100 mL of anhydrous ethyl alcohol, over which a solution of 0.02 mol of sodium monochloroacetate in 40 mL of anhydrous ethyl alcohol was gradually added with stirring. The solution was refluxed with stirring for two hours and then filtered. The filtrate was concentrated by distillation under low pressure to a volume of 45–50 mL. By cooling, the sodium salts (IIIa–IIIc) precipitate and then are separated by filtration and washing with anhydrous ethyl alcohol, previously cooled.

The thioacetates were purified by hot dissolution into 100 mL of anhydrous methyl alcohol, treatment with 5 g of activated carbon, and then hot filtration. By cooling, the product is separated from filtrate in the form of pale-yellow crystals. After filtering, the product is dried in a vacuum oven at 45–55 °C and is conditioned in a closed container.

**3-[(5′-Nitro-1H-indazol-1′-yl)-methyl]-N-phenyl-1,2,4-triazol-5-α- sodium thioacetates (IIIa)**

Yellow, crystalline solid (7.51 g; yield 87%); m.p. = 203–205 °C.

Anal. calc. for C_18_H_13_N_6_O_4_SNa (%): C, 50.00; H, 3.00; N, 19.44; S, 7.40. Found (%): C, 50.19; H, 3.31; N, 19.84; S, 7.76.

FT-IR; γ_max_ (cm^−^^1^): 1344 (NO_2_ symm); 1514 (NO_2_ assym); 1673 (C=N); 1420 (S–CH_2_); 1616 (COO^–^); 785, 811 (monosubstituted benzene nucleus).

^1^H-NMR (DMSO-d_6_, 400 MHz), δ (ppm): 4.49 (s, 2H, CH_2_); 5.61 (s, 2H, CH_2_); 7.09–7.10 (d, *J* = 6.4 Hz, 2H, CHAr); 7.19–7.21 (d, *J* = 6.4 Hz, 2H, CHAr); 7.44–7.47 (t, 1H, CHAr); 7.66–7.68 (d, *J* = 9.6 Hz, 1H, CHAr); 8.11 (s, 1H, CHAr); 8.19 (s, 1H, CHAr); 8.26–8.27 (d, *J* = 5.6 Hz, 1H, CHAr).

**3-[(5′-Nitro-1H-indazol-1′-yl)-methyl]-N-(p-tolil)-1,2,4-triazol-5-α- sodium thioacetates (IIIb)**

White-yellow, crystalline solid (7.84 g; yield 88%); m.p. = 214–216 °C.

Anal. calc. for C_19_H_15_N_6_O_4_SNa (%): C, 51.12; H, 3.36; N, 18.83; S, 7.17. Found (%): C, 51.38; H, 3.50; N, 19.16; S, 7.56.

FT-IR; γ_max_ (cm^−^^1^): 1338 (NO_2_ symm); 1519 (NO_2_ assym); 1678 (C=N); 1496 (S–CH_2_); 1617 (COO^–^); 799 (para-disubstituted benzene nucleus).

^1^H-NMR (DMSO-d_6_, 400 MHz), δ (ppm): 2.19 (s, 3H, CH_3_); 4.40 (s, 2H, CH_2_); 5.64 (s, 2H, CH_2_); 7.05–7.07 (d, *J* = 6.0 Hz, 2H, CHAr); 7.15–7.17 (d, *J* = 9.2 Hz, 1H, CHAr); 7.40–7.42 (d, *J* = 9.6 Hz, 1H, CHAr); 7.69–7.70 (d, *J* = 4.8 Hz, 1H, CHAr); 8.26 (s, 1H, CHAr); 8.79 (s, 1H, CHAr).

**3-[(5′-Nitro-1H-indazol-1′-yl)-methyl]-N-(p-methoxyphenyl)-1,2,4-triazol-5-α-sodium thioacetates (IIIc)**

Dark-yellow, crystalline solid (8.50 g; yield 92%); m.p. = 220–222 °C.

Anal. calc. for C_19_H_15_N_6_O_5_SNa (%): C, 49.35; H, 3.24; N, 18.18; S, 6.92. Found (%): C, 49.47; H, 3.62; N, 18.47; S, 7.33.

FT-IR; γ_max_ (cm^−^^1^): 1346 (NO_2_ symm); 1515 (NO_2_ assym); 1675 (C=N); 1421 (S–CH_2_); 1067 (Ar–OCH_3_); 1617 (COO^–^); 820 (para-disubstituted aromatic nucleus).

^1^H-NMR (DMSO-d_6_, 400 MHz), δ (ppm): 3.39 (s, 3H, OCH_3_); 4.38 (s, 2H, CH_2_); 5.67–5.71 (d, *J* = 15.6 Hz, 2H, CH_2_); 7.00–7.02 (d, *J* = 10.0 Hz, 2H, CHAr); 7.03–7.04 (d, *J* = 5.6 Hz, 2H, CHAr); 7.53–7.55 (d, *J* = 11.2 Hz, 1H, CHAr); 8.05–8.06 (d, *J* = 2.4 Hz, 1H, CHAr); 8.31 (s, 1H, CHAr); 8.36 (s, 1H, CHAr).

**3-[(5′-Nitro-1H-indazol-1′-yl)-methyl]-N-aryl-1,2,4-triazol-5-thioacetamido-sodium penicillins (IVa–IVc)**

The 6-aminopenicillanic acid (6-AP) triethylammonium salt solution was prepared by stirring at room temperature, for 60 min, 0.015 mol 6-AP acid and 0.015 mol triethylamine in 50 mL anhydrous dichloromethane.

Separately, mixed pivalic anhydride was prepared: 0.015 mol derived from 1,2,4-triazole-5-α-sodium thioacetate (IIIa–IIIc) was dissolved in 50 mL of anhydrous dichloromethane, cooled at 0–5 °C, and 0.015 mol of freshly distilled pivalic chloride was added under stirring.

Next, the 6-AP acid triethylammonium salt solution was added to the mixed anhydride, and stirring was continued at 8–10 °C for 60 min. After stopping the cooling, the reaction mixture was stirred for two hours, during which the working temperature reached 20–25 °C. The dichloromethane solution was removed by vacuum distillation below 25 °C and the remaining residue was dissolved in 250 mL of saturated bicarbonate solution. The alkaline aqueous solution thus obtained was acidified with dilute hydrochloric acid (1:1) to pH = 1.5–2.0, in the presence of 50 mL of butyl acetate. The acidic penicillin passes into the organic layer which, in order to remove traces of hydrochloric acid, was washed twice with 25 mL of distilled water. The washed butyl acetate was dried over 15 g of anhydrous sodium sulphate. After filtration, the sodium sulphate was washed twice with 15 mL of anhydrous butyl acetate each time.

The sodium salt of the formed penicillin was isolated by treating the combined solutions of butyl acetate with 0.015 mol of sodium acetate, under stirring at room temperature. Within three hours, the precipitation of sodium penicillins (IVa–IVc) was complete.

The obtained monocrystals were filtered and washed on a filter with anhydrous acetone. The purification of penicillins is performed by dissolving them in the form of sodium salts in distilled water, acidifying them in the presence of butyl acetate, and processing the organic layer in the same way as described above.

The purification of the penicillins (IVa–IVc) was achieved by dissolving them in water and extracting the acid penicillin after acidulation, followed by the re-precipitation of sodium salts. During re-precipitation, the sodium acetate was used in smaller amounts than is theoretically required. The obtained microcrystals were filtered and washed on the filter with acetone. After drying, the penicillins were dosed by iodometric method, while the elemental analysis, spectral analysis (FT-IR, ^1^H-NMR, ^13^C-NMR), and optical rotation were measured. The purification was applied until a constant value of optical rotation was obtained: 173.0° for IVa, 176.0° for IVb, and 187.0° for IVc, respectively. The percentage content of penicillins IVa–IVc was determined by iodometric method: 99.4% for IVa, 99.7% for IVb, and 99.9% for IVc.

**3-[(5′-Nitro-1H-indazol-1′-yl)-methyl]-N-phenyl-1,2,4-triazol-5-thioacetamido-sodium penicillins (IVa)**

Light-grey, crystalline solid (7.37 g; yield 78%); m.p. = 137–138 °C.

Anal. calc. for C_26_H_23_N_8_O_6_S_2_Na (%): C, 49.52; H, 3.65; N, 17.77; S, 10.15. Found (%): C, 49.69; H, 3.85; N, 18.13; S, 10.42.

FT-IR; γ_max_ (cm^−^^1^): 1348 (NO_2_ symm); 1515 (NO_2_ assym); 3115 (NH amide); 1616 (CO amide); 1790 (CO β-lactam); 1417 (C–S–C); 1065 (C–N third); 1610 (COO^–^); 816 (monosubstituted benzene nucleus).

^1^H-NMR (DMSO-d_6_, 400 MHz), δ (ppm): 1.51 (s, 6H, 2CH_3_); 3.79 (s, 2H, CH_2_); 4.15–4.57 (m, 3H, 3CH); 5.22 (s, 2H, CH_2_); 7.15–7.16 (d, *J* = 2.0 Hz, 2H, CHAr); 7.28–7.30 (d, *J* = 7.6 Hz, 2H, CHAr); 7.55–7.58 (t, 1H, CHAr); 7.80–7.81 (d, *J* = 3.6 Hz, 1H, CHAr); 8.19 (s, 1H, CHAr); 8.23 (s, 1H, CHAr); 8.29–8.30 (d, *J* = 3.2 Hz, 1H, CHAr); 9.02 (s, 1H, NH).

^13^C-NMR (DMSO-d_6_, 100 MHz), δ (ppm): 41.32; 54.70 (CH_2_); 111.51; 119.42; 123.56; 131.61; 133.57 (Ar); 98.66; 134.29; 146.19; 147.60; 148.34; 148.79 (C-N); 48.46 (C-H β-lactam ring); 28.79 (CH_3_); 66.31; 145.39 (C-S); 171.98; 172.42; 177.87 (C=O).

**3-[(5′-Nitro-1H-indazol-1′-yl)-methyl]-N-(p-tolil)-1,2,4-triazol-5-thioacetamido-sodium penicillins (IVb)**

White, crystalline solid (7.91 g; yield 82%); m.p. = 142–145 °C.

Anal. calc. for C_27_H_25_N_8_O_6_S_2_Na (%): C, 50.31; H, 3.88; N, 17.39; S, 9.93. Found (%): C, 50.64; H, 4.07; N, 17.76; S, 10.30.

FT-IR; γ_max_ (cm^−^^1^): 1373 (NO_2_ symm); 1534 (NO_2_ assym); 3312 (NH amide); 1635 (CO amide); 1735 (CO β-lactam); 1418 (C–S–C); 1187 (C–N third); 1596 (COO^–^); 765 (para-disubstituted benzene nucleus).

^1^H-NMR (DMSO-d_6_, 400 MHz), δ (ppm): 1.49 (s, 6H, 2CH_3_); 2.11–2.14 (d, *J* = 13.2 Hz, 3H, CH_3_); 3.93 (s, 2H, CH_2_); 4.23–4.41 (m, 3H, 3CH); 5.16 (s, 2H, CH_2_); 7.18–7.20 (d, *J* = 10.0 Hz, 2H, CHAr); 7.30–7.32 (d, *J* = 8.8 Hz, 2H, CHAr); 7.49–7.52 (t, 1H, CHAr); 7.77–7.79 (d, *J* = 6.8 Hz, 1H, CHAr); 8.22 (s, 1H, CHAr); 8.31 (s, 1H, CHAr); 8.35–8.36 (d, *J* = 3.2 Hz, 1H, CHAr); 9.13 (s, 1H, NH).

^13^C-NMR (DMSO-d_6_, 100 MHz), δ (ppm): 25.62; 28.89 (CH_3_); 42.09; 54.83 (CH_2_); 111.85; 119.44; 123.97; 131.20; 133.84; 136.12 (Ar); 97.52; 139.35; 143.92; 145.36; 147.39; 147.91 (C-N); 47.89 (C-H β-lactam ring); 65.79; 145.80 (C-S); 171.83; 172.59; 177.30 (C=O).

**3-[(5′-Nitro-1H-indazol-1′-yl)-methyl]-N-(p-methoxyphenyl)-1,2,4-triazol-5-thioacetamido-sodium penicillins (IVc)**

White, crystalline solid (8.51 g; yield 86%); m.p. = 127–130 °C.

Anal. calc. for C_27_H_25_N_8_O_7_S_2_Na (%): C, 49.09; H, 3.78; N, 16.96; S, 9.69. Found (%): C, 49.35; H, 3.99; N, 17.29; S, 10.06.

FT-IR; γ_max_ (cm^−^^1^): 1337 (NO_2_ symm); 1520 (NO_2_ assym); 1067 (Ar–OCH_3_); 3120 (NH amide); 1677 (CO amide); 1702 (CO β-lactam); 1497 (C–S–C); 1259 (C–N third); 1618 (COO^–^); 798 (para-disubstituted benzene nucleus).

^1^H-NMR (DMSO-d_6_, 400 MHz), δ (ppm): 1.44 (s, 6H, 2CH_3_); 3.71 (s, 2H, CH_2_); 3.98 (s, 3H, OCH_3_); 4.16–4.59 (m, 3H, 3CH); 5.41 (s, 2H, CH_2_); 7.13–7.14 (d, *J* = 4.8 Hz, 2H, CHAr); 7.26–7.31 (d, *J* = 22.0 Hz, 2H, CHAr); 7.89–7.92 (d, *J* = 11.6 Hz, 1H, CHAr); 8.22 (s, 1H, CHAr); 8.24 (s, 1H, CHAr); 8.44–8.45 (d, *J* = 4.4 Hz, 1H, CHAr); 9.17 (s, 1H, NH).

^13^C-NMR (DMSO-d_6_, 100 MHz), δ (ppm): 29.10; 56.79 (CH_3_); 42.20; 54.61(CH_2_); 111.68; 119.52; 124.02; 131.73; 137.51; 148.13 (Ar); 91.56; 139.55; 143.37; 145.29; 147.13; 147.58 (C-N); 49.18 (C-H β-lactam ring); 65.35; 144.86 (C-S); 159.79; 171.33; 172.47; 177.82 (C=O).

### 3.3. Biological Activity Tests

The acute toxicity was established by evaluation of produced mortality. The toxicity degree indicates the maximum bearable dose *LD*_0_, minimum lethal dose *LD*_100_, and the killing dose that kills 50% of the experimental animals *LD*_50_, as these represent the best clues in the interpretation of the results. In the determination of the acute toxicity of the compounds, mice weighing 20 ± 2 g were used, the experimental groups consisting of 10 animals of both sexes. The animals were kept under observation for 7 days at room temperature (22 °C ± 1 °C), receiving standard food and water. Their weight was determined by periodic weighing every two days at the same time. The animals that lost weight were removed. The substances were administered intraperitoneally as a suspension in Tween 80 and the mortality was recorded at 14 days. The Spearman–Kärber method [27] allowed for the quick determination of *LD*_50_ on a small number of animals, using the calculation method:

(1) $$LD_{50}=LD_{100}-\sum \frac{a\times b}{n},$$

where *a* is the difference between two successive doses of the administrated substance, *b* is the average number of dead animals in two successive doses, and *n* is the total number of animals in the group.

This study was conducted according to the guidelines of the Declaration of Helsinki and approved by the Ethics Committee of the Faculty of Veterinary Medicine, “Ion Ionescu de la Brad” University of Agricultural Sciences and Veterinary Medicine of Iasi, Romania (protocol code 220 from 24 June 2021).

The gram-positive and gram-negative germs were prepared in advance, regenerating from the storage medium, after which their morphological, tincture, and culture characteristics were verified: solids Mueller–Hinton, blue agar-bromothymol-lactose, agar-blood, as well as on the liquid medium nutritious simple meat broth.

The metabolic characteristics were monitored on the mediums Triple-Sugar-Iron agar, Mobility-Indole-Urea, Motility-Indole-Lysine Decarboxylase, and Phenylalanine deaminase and confirmed by APJ-20-Easystem kits.

All tested strains showed fully expressed phenotypic characteristics, being pure and qualitatively suitable for use in the experiment.

Bacterial cultures were prepared for testing using suspensions with a concentration of 5.2 × 10^7^ colony forming units/mL, density 0.5 McFarland, determined with the densimeter.

Both gram-positive germs *Streptococcus pneumoniae* (ATTC-46619), *Staphylococcus aureus* (ATCC-25923) and gram-negative germs *Klebsiella pneumoniae* (C-82), *Pseudomonas aeruginosa* (ATCC-27853), *Escherichia coli* (ATCC-25922) were used to test the antimicrobial activity of the newly obtained semisynthetic penicillins (IVa–IVc).

Amoxicillin, known for its antimicrobial activity against certain gram-positive and gram-negative germs, was used as a reference substance.

The microbial strains, as a suspension in saline, were cultured on Mueller–Hinton agar and incubated at 37 °C for 18 h.

The newly obtained semisynthetic penicillins were dissolved in Tween 80 (100 μg/mL Tween 80), after which the resulting solutions were introduced into the culture medium (100 mL/sample). The same was conducted with the reference antibiotic (amoxicillin). Sterilization was performed at 120 °C for 20 min. In parallel, a control test was performed only with Tween 80, to highlight the possible activity of the solvent on the test germs.

All operations were performed in the sterile box. The assessment of antimicrobial activity was performed by the Kirby–Bauer method [31], the readings being made at 24 h.

The minimum inhibitory concentration of the penicillins IVa–IVc was determined by the broth microdilution method [32] from the stock solution (100 μg antibiotic/mL Tween 80), obtaining 12 diluted solutions with different concentrations, which were introduced in the culture medium. The microdilution plates were fitted with a tight lid and kept at 37 °C for 18 h before interpreting the results, except for *Streptococcus pneumoniae*, which was kept for 20 h. These tests were carried out in the Microbiology Laboratory, Epidemiology section of the Institute of Public Health from Iasi, Romania.

## 4. Conclusions

This research is in a field that addresses the extent of the acylation reaction of 6-aminopenicillanic acid with new triazole derivatives.

Using sodium 5-thioacetates of 1,2,4-triazoles-3,4-disubstituted as acylating agents of 6-aminopenicillanic acid, by the method of mixed anhydrides—the activating partner being pivaloyl chloride—a series of new semisynthetic penicillins were prepared from which important therapeutic effects can be expected.

The intermediates and final synthesis products were characterized by elemental and spectral analysis (FT-IR, ^1^H- and ^13^C-NMR). Data from chemical analysis and spectrometric measurements rigorously confirm all structural details.

The synthesized penicillins were studied in terms of toxicological action and antimicrobial activity, finding promising results, some of them showing biological activity comparable to the reference drug in circulation (amoxicillin). Moreover, the penicillins IVb and IVc are active also against the gram-negative germ *Pseudomonas aeruginosa*.

## Figures and Tables

**Table 1:** Test data for determination of *LD*_50_ values for penicillin IVa (*n*—number of mice in each group, *a*—difference between two consecutive doses, and *b*—average value of the deaths from two consecutive doses).

Dose (mg/kg Body Weight)	Death	*n*	*a* (mg/kg Body Weight)	*b*	*ab*
3000	0	10	1000		
4000	3	10	1000	1.5	1500
5000	6	10	1000	4.5	4500
6000	7	10	1000	6.5	6500
7000	9	10	1000	8	8000
8000	10	10	1000	9.5	9500

**Table 2:** Test data for determination of *LD*_50_ values for penicillin IVb (*n*—number of mice in each group, *a*—difference between two consecutive doses, and *b*—average value of the deaths from two consecutive doses).

Dose (mg/kg Body Weight)	Death	*n*	*a* (mg/kg Body Weight)	*b*	*ab*
3000	0	10	1000		
4000	3	10	1000	1.5	1500
5000	5	10	1000	4	4000
6000	7	10	1000	6	6000
7000	9	10	1000	8	8000
8000	10	10	1000	9.5	9500

**Table 3:** Test data for determination of *LD*_50_ values for penicillin IVc (*n*—number of mice in each group, *a*—difference between two consecutive doses, and *b*—average value of the deaths from two consecutive doses).

Dose (mg/kg Body Weight)	Death	*n*	*a* (mg/kg Body Weight)	*b*	*ab*
3000	0	10	1000		
4000	2	10	1000	1	1000
5000	5	10	1000	3.5	3500
6000	7	10	1000	6	6000
7000	9	10	1000	8	8000
8000	10	10	1000	9.5	9500

**Table 4:** Test data for determination of *LD*_50_ values for amoxicillin (*n*—number of mice in each group, *a*—difference between two consecutive doses, and *b*—average value of the deaths from two consecutive doses).

Dose (mg/kg Body Weight)	Death	*n*	*a* (mg/kg Body Weight)	*b*	*ab*
3000	0	10	1000		
4000	2	10	1000	1	1000
5000	4	10	1000	3	3000
6000	7	10	1000	5.5	5500
7000	8	10	1000	7.5	7500
8000	10	10	1000	9	9000

**Table 5:** *LD*_50_ values for penicillins IVa—IVc and amoxicillin as reference drug.

Compound	*LD*_50_ (mg/kg Body Weight)
IVa	5000
IVb	5100
IVc	5200
Amoxicillin	5400

**Table 6:** Antimicrobial activity of the penicillins IVa–IVc and amoxicillin as reference drug.

Compound	Diameter of the Inhibition Zone (mm)				
	*Streptococcus pneumoniae*	*Staphylococcus aureus*	*Klebsiella pneumoniae*	*Pseudomonas aeruginosa*	*Escherichia coli*
IVa	6–7	7–8	8–9	10–11	11–12
IVb	14–15	13–14	16–17	18–19	17–18
IVc	21–22	23–24	24–25	26–27	29–30
Amoxicillin	25–26	30–31	26–27	–	34–35

**Table 7:** Minimum inhibitory concentration of the penicillins IVb and IVc with amoxicillin as reference drug.

Compound	Minimum Inhibitory Concentration (μg/mL)				
	*Streptococcus pneumoniae*	*Staphylococcus aureus*	*Klebsiella pneumoniae*	*Pseudomonas aeruginosa*	*Escherichia coli*
IVb	1.5	7.0	16.0	10.0	15.0
IVc	0.7	5.0	9.5	9.0	8.5
Amoxicillin	0.5	4.0	8.0	-	8.0

## Data Availability

The data presented in this study are available on request from the corresponding author.
